# A fatal combination of situs inversus, pregnancy and cardiac arrest treated with an automated external defibrillator

**DOI:** 10.1007/s12471-016-0851-5

**Published:** 2016-05-23

**Authors:** S. Calle, M. De Leeuw, N. Mpotos, P. Calle, B. De Turck

**Affiliations:** 1 Faculty of Medicine and Health Sciences, Ghent University, Ghent, Belgium; 2 Forensic Pathology Department, Ghent University Hospital, Ghent, Belgium; 3 Department of Emergency Medicine, Sint Lukas General Hospital, Ghent, Belgium; 4 Faculty of Medicine and Health Sciences, University of Antwerp, Wilrijk, Belgium; 5 Department of Emergency Medicine, Maria Middelares General Hospital, Ghent, Belgium

A 34-year-old female suddenly collapsed and remained comatose. She was 6 months pregnant. Information on previous medical problems could not be obtained, due to a language barrier. Upon arrival of the first tier ambulance she was unresponsive with a pulse of 30 beats/min. A few minutes later, no pulse could be detected and basic life support was started with an automated external defibrillator (AED). Self-adhesive pads were placed in the conventional sternal-apical position. The first and second rhythm analyses led to a no-shock decision (Fig. 1). The third and fourth analyses gave rise to shocks (Fig. 2). The patient was transferred to the hospital with ongoing advanced cardiac life support and taken to the delivery room for caesarean section. Maternal and newborn resuscitation were unsuccessful. Forensic autopsy revealed situs inversus, but no apparent cause of death [1–3].

**Fig. 1:**
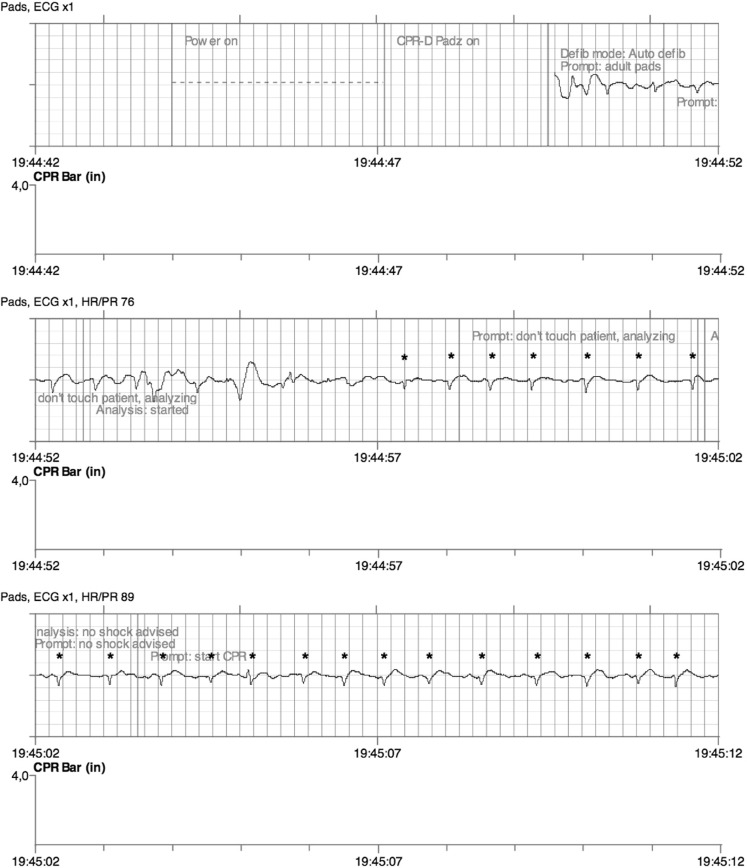
The decision of the rhythm analysis algorithm on the first recorded rhythm by the automated external defibrillator was ‘no shock’. This decision was judged to be correct by the presence of small QRS complexes at a rate of approximately 85 per minute (marked with asterisks). Note that during this analysis there were some minor external artefacts between 19:44:53 and 19:44:56, but no chest compressions.

**Fig. 2:**
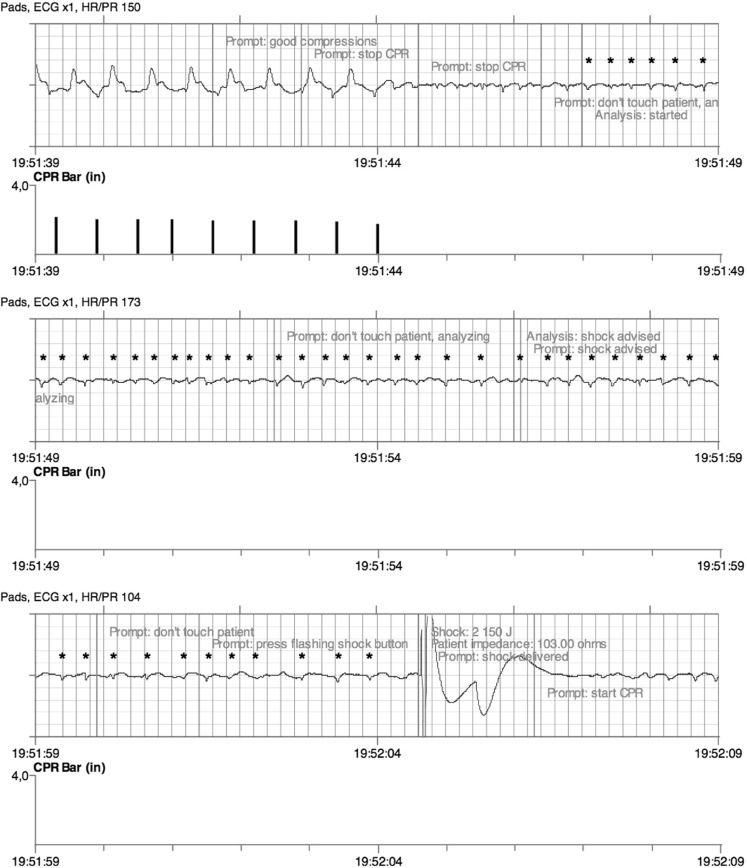
The fourth analysis (started at 19:51:47) gave rise to ‘shock advised’ (at 19:51:56) and the delivery of a shock (at 19:52:04). This decision was judged to be wrongful as there are still the same small QRS complexes (marked with asterisks) as in Fig. 1. The higher rate of the QRS complexes is related to the resuscitation attempt including epinephrine administration. Note that there were no chest compressions during the analysis; as shown by the marks under ‘CPR bar’, chest compressions were halted at 19:51:44.

What do you think of the shock/no-shock decisions by the AED?

## Answer

You will find the answer elsewhere in this issue.
